# Revealing anelasticity and structural rearrangements in nanoscale metallic glass films using *in situ* TEM diffraction

**DOI:** 10.1080/21663831.2016.1228709

**Published:** 2016-09-22

**Authors:** Rohit Sarkar, Christian Ebner, Ehsan Izadi, Christian Rentenberger, Jagannathan Rajagopalan

**Affiliations:** ^a^ Department of Materials Science and Engineering, School for Engineering of Matter, Transport and Energy, Arizona State University, Tempe, AZ, USA; ^b^ Physics of Nanostructured Materials, Faculty of Physics, University of Vienna, Vienna, Austria; ^c^ Department of Mechanical and Aerospace Engineering, School for Engineering of Matter, Transport and Energy, Arizona State University, Tempe, AZ, USA

**Keywords:** Metallic glass, atomic rearrangements, local elastic strain, *in situ* electron diffraction, strain rate experiments

## Abstract

We used a novel diffraction-based method to extract the local, atomic-level elastic strain in nanoscale amorphous TiAl films during *in situ* transmission electron microscopy deformation, while simultaneously measuring the macroscopic strain. The complementary strain measurements revealed significant anelastic deformation, which was independently confirmed by strain rate experiments. Furthermore, the distribution of first nearest-neighbor distances became narrower during loading and permanent changes were observed in the atomic structure upon unloading, even in the absence of macroscopic plasticity. The results demonstrate the capability of *in situ* electron diffraction to probe structural rearrangements and decouple elastic and anelastic deformation in metallic glasses.

## GRAPHICAL ABSTRACT

**Figure:**
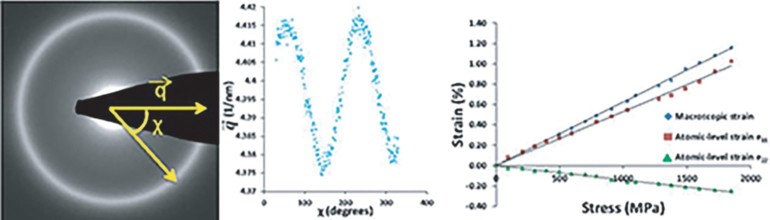

**IMPACT STATEMENT**

This paper employs a novel *in situ* electron diffraction technique to reveal deformation-induced structural rearrangements, and decouple atomic-level elastic strain from larger scale anelastic strain in metallic glasses.

## 1. Introduction

Metallic glasses have higher strength, hardness and corrosion resistance than conventional metals and better toughness than ceramics, making them highly attractive for a variety of applications [1–3]. In spite of their limited ductility, metallic glasses have shown promise for structural applications due to their high elastic limit, which can extend up to 2% [1,4]. Nevertheless, significant efforts have been directed toward addressing the lack of ductility, which is attributed to highly localized plastic flow in shear bands [4]. In one such study, Wang et al*.* showed that enhanced plastic deformation in Zr-based bulk metallic glasses is possible through the suppression of shear localization by using geometrical constraints [5]. Alternatively, researchers have shown that when sample dimensions are reduced to the nanoscale, homogeneous flow supersedes shear band propagation as the dominant mode of deformation and results in enhanced ductility [6,7]. Furthermore, molecular dynamics (MD) simulations have revealed that metallic glass films can deform plastically below a critical thickness by non-localized flow [8], providing further promise for increased ductility at the nanoscale.

In addition to enhancing ductility, numerous studies have also focused on understanding the deformation processes in metallic glasses, which are notably different from conventional metals due to the absence of long-range order and defects such as dislocations and grain boundaries [1,9–14]. In particular, *in situ* high-energy X-ray and neutron diffraction techniques have been extensively used to measure micro strains in metallic glass systems [15–18]. These techniques calculate the elastic micro strain tensor by measuring the relative shift of diffraction peaks in reciprocal space during straining. Such *in situ* deformation studies have helped to shed new light into the mechanical behavior of metallic glasses. For instance, it has been shown that micro strains in metallic glasses are dependent on length scales [19], that is, nearest-neighbor shells are stiffer compared to distant atomic shells, which is contrary to the conventional understanding that the bonds in metallic glasses are isotropic.

Several other insights on the elastic and plastic properties of metallic glasses have also emerged from such studies. Ma et al*.* used *in situ* neutron diffraction to show that the Young's and shear modulus of various metallic glasses are similar to their base metal component [20]. Vempati et al*.* have postulated that the length scale dependence of strain in metallic glasses is due to heterogeneous non-affine atomic displacements [19]. Scudino et al. have found that shear strain and structural anisotropy play a major role in the plastic behavior of metallic glasses [21]. High-energy X-ray scattering has also been employed to study the strain around crack tips in metallic glasses [22] and the mechanism of nucleation and propagation of shear bands during loading [23]. While these studies have significantly enhanced our understanding of the deformation processes in metallic glasses, the use of X-ray or neutron diffraction has restricted the experiments to bulk, macroscopic samples. Therefore, the deformation behavior of metallic glasses at the micro and nanoscale has remained relatively unexplored.

In this study, a novel technique [24] was used to extract the local 2D elastic strain tensor from selected area electron diffraction (SAD) patterns during *in situ* transmission electron microscopy (TEM) tensile straining of freestanding nanoscale TiAl metallic glass films. The atomic-level elastic strain was derived by analyzing the deformation-induced anisotropic geometric changes in the first diffuse ring of the SAD patterns. The *in situ* TEM straining was enabled by micro-electro-mechanical systems (MEMS)-based tensile testing stages that allow the concurrent measurement of the macroscopic stress and strain (*ε_macro_*_­_) on the thin film samples. The *in situ* experiments showed that the macroscopic stress–strain response was linear. However, the local elastic strain derived from the SAD patterns was consistently lower than *ε_macro_*_­_, revealing the presence of anelastic deformation. This anelasticity was independently confirmed by measuring the rate dependent stress–strain response of the film. In addition, the straining narrowed the spread of nearest-neighbor atomic distances as revealed by a reduction in the width of the SAD amorphous ring.

The results show that *in situ* TEM electron diffraction can be used to detect deformation-induced structural rearrangements, and decouple atomic-level elastic strain from anelastic deformation in metallic glasses. A unique advantage of this technique is that elastic strain can be measured at precise locations with sub-micrometer resolution, which is not possible using X-ray or neutron diffraction. This capability, for example, could be used to measure elastic strains near crystallites in a partially devitrified metallic glass. These crystallites have been shown to alter the fracture morphology [25], and an accurate measurement of their near-field elastic strain can lead to a more quantitative understanding of this behavior. In addition to the above, loading induced microstructural changes can potentially be monitored through special imaging techniques to obtain a more comprehensive picture of the deformation processes in metallic glasses.

## 2. Experimental details

### 2.1. Fabrication of TiAl films and MEMS devices

**Figure 1.**
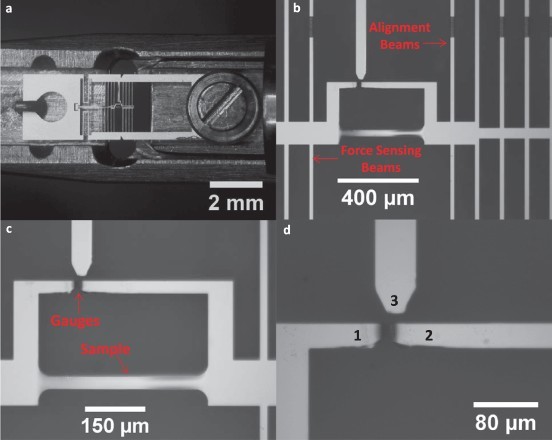
MEMS device for *in situ* TEM straining: (a) Optical micrograph of a typical MEMS device mounted on the Philips TEM straining holder. (b) A magnified image of the device showing the alignment beams that ensure nearly perfect uniaxial loading of the sample and force-sensing beams which are used to measure the macroscopic stress on the sample. (c) Image showing the freestanding film sample and force- and strain-sensing gauges. (d) The change in distance between gauges 1 and 2 gives the macroscopic deformation of the sample, while the relative deflection of gauge 1 with respect to the stationary gauge 3 multiplied by the stiffness of the force-sensing beams gives the force acting on the sample.

A 150-nm-thick TiAl (45 atomic % Ti, 55 atomic % Al) film was synthesized by the co-deposition of Ti and Al on a 4′′ diameter, 200-µm-thick, (100)-oriented silicon wafer by DC Magnetron Sputtering at a base pressure of 5 × 10^−8^ Torr. The composition of the film was controlled by varying the power on the individual sputtering guns containing 99.999% pure Ti and Al targets. The film was amorphous in the as-deposited state. Rutherford backscattering spectrometry (RBS) was used to determine the composition of the films and X-ray diffraction (XRD) analysis confirmed the amorphous nature of the film. Photolithography and reactive ion etching techniques were then used to co-fabricate MEMS-based tensile testing devices having built-in strain and force gauges along with dog-bone-shaped freestanding film samples (Figure 1). The device is designed such that nearly perfect uniaxial tensile loading is applied on the thin film samples, and finite element analysis has shown that the device reduces any potential misalignment by six orders of magnitude [26]. A detailed description of the MEMS devices and the process used for their fabrication can be found in [27,28]. The MEMS devices were 2.5 mm wide and 9 mm long, while the freestanding film samples had an effective gauge length of 395 µm and a width of 30 µm.

### 2.2. *In situ* and *ex situ* tensile testing procedure

For the *in situ* TEM experiments, MEMS devices containing the TiAl film samples were loaded onto a Philips™ straining TEM holder and tensile tests were carried out in a Philips CM200 TEM at an accelerating voltage of 200 kV. The samples were uniaxially strained in steps of 150 nm and bright-field images and SAD patterns were recorded using a Gatan™ Orius CCD camera after allowing the film to relax for 5 min. The averaged strain rate over the duration of the experiments was ∼10^−6^/s, essentially resulting in quasi-static loading.

SAD patterns were taken from a circular area of diameter 1.2 μm with an exposure time of 10 s. Before each SAD pattern was acquired, a normalization procedure was performed to reduce the magnetic remanence of the lenses so that the variation of the camera length in different SAD patterns was minimized. The illumination condition of the TEM was also kept constant during the experiment. To ensure that the electron beam did not cause any stress relaxation or anomalous changes in sample geometry[29], we continuously exposed one of the samples to the electron beam for 60 min and recorded the stress, *ε_macro_*_­_ and atomic-level elastic strain before and after exposure. No noticeable difference was seen in any of these quantities.

Three TiAl film samples were tested *in situ* in the TEM. The first sample was loaded up to *ε_macro_*_­_ = 0.6%, while the second and third samples were loaded until failure. The second sample was loaded and unloaded once before it was strained to failure, whereas the first and third samples were subjected to a single loading. In addition, *ex situ* uniaxial tensile load–unload experiments were performed at different strain rates (10^−6^/s to 10^−2^/s) on two samples. A piezoelectric actuator (Physik Instrumente) was used to load the MEMS devices and a CMOS camera (Thor Labs) was used to acquire images of the gauges during the experiments. A more detailed description of the procedure for these strain rate experiments is provided in [30].

### 2.3. Analysis of macroscopic and atomic-level elastic strains

**Figure 2.**
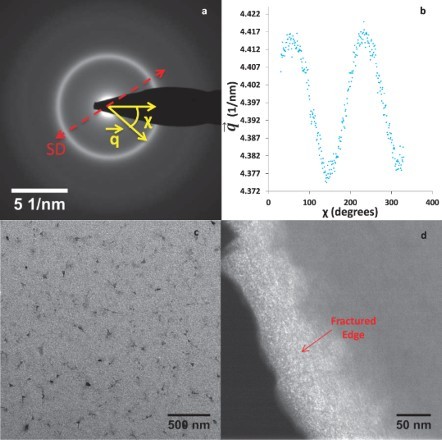
Evolution of the SAD pattern ellipticity and microstructure of TiAl film: (a) SAD pattern of the amorphous TiAl film, where 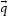 corresponds to the reciprocal lattice vector of the first ring and *χ* is the azimuthal angle. The dotted red arrow indicates the straining direction (SD), which is along the length of the sample. SD is not aligned with the *χ* = 0 direction due to the rotation induced by the magnetic lenses when switching from imaging mode to diffraction mode. (b) A plot of the reciprocal lattice vector (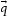) corresponding to the maxima positions of the SAD ring shows the peaks and valleys appearing at different azimuthal angles (*χ*) due to the ellipticity introduced by straining. (c) TEM bright-field image of the TiAl film before straining, indicative of a typical amorphous microstructure. (d) TEM dark-field image of the fractured edge of the third sample. The objective aperture was placed on the first ring of highest intensity to obtain the dark-field image. The brightness gradient across the fractured edge indicates a change in sample thickness and suggests failure by shear localization.

The macroscopic stress and strain on the samples were measured using the built-in gauges (Figure 1(c)) of the MEMS stage. A custom MATLAB^TM^ program, which tracks prescribed features across a series of images using cross-correlation techniques, was used to measure the displacement of the gauges, and thus the sample stress and strain. The macroscopic stress and strain resolutions were 2 MPa and 0.005%, respectively, for the *in situ* TEM experiments and 5 MPa and 0.01%, respectively, for the *ex situ* strain rate experiments. A script [24] written on the GATAN™ Digital-Micrograph platform was used to analyze the SAD patterns and extract the 2D strain tensor and the principal strain along the longitudinal (*e_11_*) and transverse directions (*e_22_*). The script measures the ellipticity introduced in the SAD ring by deformation to extract the elastic strain (Figure 2(a)-(b)).

Since these elastic strains correspond to the strain at the atomic level, we denote them as atomic-level elastic strains to distinguish them from the macroscopic strain (*ε_macro_*_­_) of the entire sample. The atomic-level elastic strain can be quantified on a micrometer scale using this technique with accuracy and precision of 10^−4^ and 2 × 10^−4^, respectively [24].

## 3. Results

### 3.1. Microstructure and composition of the film

TEM bright-field images of the film showed contrast that is characteristic of a metallic glass, containing no crystalline phases (Figure 2(c)). It should be pointed out that the dark features present in the bright-field images are photoresist residues left from sample fabrication. The amorphous structure of the thin film was confirmed by the SAD pattern containing broad diffuse rings (Figure 2(a)) as well as XRD. RBS measurements revealed the composition of the film to be 45 atomic % Ti and 55 atomic % Al.

### 3.2. Stress–strain response from *in situ* TEM experiments

**Figure 3.**
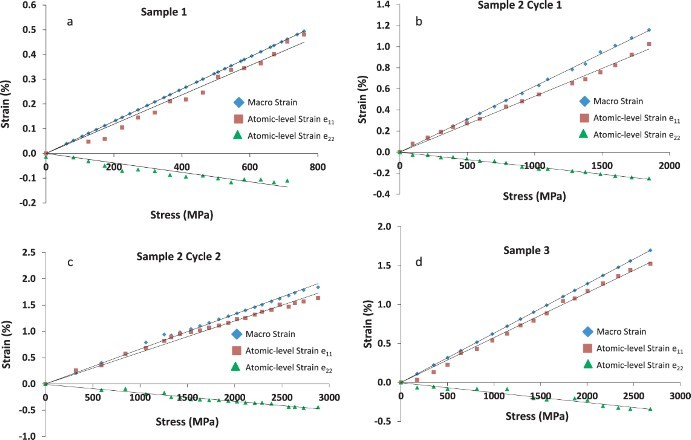
Macroscopic and atomic-level elastic strain versus stress: (a) The macroscopic and atomic-level elastic strains versus stress plot for the first TiAl sample. (b) Strain versus stress plot for the first cycle of the second sample. The sample was unloaded before failure. (c) Strain versus stress plot of the second sample for the second cycle, where it was loaded until failure. (d) Strain versus stress plot of the third sample, which was loaded until failure. In all the plots, the black lines represent the lines of best fit, whereas the symbols represent raw data points. The error in stress measurement is ±2 MPa. The error in macroscopic strain measurement is ±0.005%, whereas the error in atomic-level elastic strain measurements is ±0.01%.

The *in situ* TEM tensile experiments revealed an absence of plasticity (Figure 3), with all three films exhibiting a linear macroscopic stress–strain behavior up to the maximum strain/failure. The failure occurred in a catastrophic manner and the fracture surface was inclined with respect to the cross section of the film, creating a wedge-shaped structure. The variation in thickness along the wedge creates a brightness gradient in the image (Figure 2(d)). From intensity measurements across the fracture surface, we calculated an inclination angle of 45–60 degrees between the fracture plane and the film surface. This is close to the typical 45-degree inclination observed for shear bands in tensile samples [31], which corresponds to the plane of maximum shear stress. Notably, in contrast to previous reports [32], bright-field, dark-field and SAD images of the TiAl fracture surface did not indicate the formation of nanocrystals by the localized deformation.

The calculated longitudinal principal strain *e_11_* correlated well with *ε_macro_*_­_ in all three samples, but it was on average about 10% smaller, leading to a similar difference in the calculated Young’s modulus (*E*). Specifically, *E* calculated using the applied stress and *ε_macro_*_­_ was found to be 155 ± 3 GPa, which is lower than the *E* of bulk crystalline TiAl (175–188 GPa) [33]. This lower *E* is consistent with the presence of anelastic deformation that is common in many metallic glasses [34]. The Young's modulus computed using the longitudinal atomic-level elastic strain (*e_11_*) values, in contrast, was found to be 173 ± 3 GPa, very similar to that of crystalline TiAl.

The Poisson's ratio (*µ*) calculated from the linear fit of the variation of *e_22_* and *e_11_* from all three samples was found to be 0.215 ± 0.02, in good agreement with the results from other metallic glass systems containing Ti and Al [1,33,35,36]. The *E* and *µ* obtained from each *in situ* TEM experiment are listed in Table 1. We also independently calculated the Poisson's ratio from *in situ* scanning electron microscopy (SEM) tensile experiments. In the *in situ* SEM experiments, we tracked the distances between markers (photoresist residue left from fabrication) along the longitudinal and transverse directions of a sample as a function of applied strain (Supplementary Figure 1). By measuring the change in the transverse strain as a function of the longitudinal strain, we obtained a Poisson's ratio of 0.23 ± 0.035, consistent with the *in situ* TEM measurements.

**Table 1.** Young's modulus (*E*) for *in situ* TEM straining experiments calculated using *ε_macro_*_­_ and *e_11_*. For sample 2, the values reflect the average of two cycles.

Sample	*E* using *ε_macro_* (GPa)	*E* using *e_11_* (GPa)	
1	154	169	0.195
2	152	176	0.245
3	158	173	0.210

### 3.3. Full width at half maximum measurements

**Figure 4.**
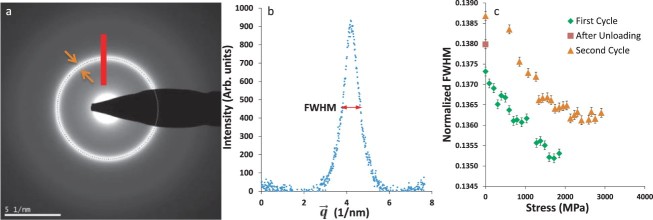
FWHM measurements on metallic glass TiAl films: (a) A typical SAD pattern analyzed during the loading of Sample 2. The orange arrows indicate the width of the first amorphous ring, which corresponds to the spread in the first nearest-neighbor atomic distances. (b) The background subtracted intensity plot corresponding to the red line in (a). The FWHM of the peak was found to change with loading. (c) The normalized FWHM values of the first diffraction ring of the second sample during the two loading cycles. The normalized FWHM values decrease with increasing stress, indicating a reduction in the spread of nearest-neighbor distances and an increase in short-range order. This change is reversed during unloading and the initial FWHM value for the 2nd cycle is higher.

We calculated the full width at half maximum (FWHM) of the first diffraction ring (Figure 4) along all directions for the samples. We then averaged the FWHM measurements and normalized it by the reciprocal lattice vector (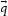) value corresponding to the zero strain direction (*χ*∼87^o^) of the elliptic diffraction ring. The normalization was done to mitigate any effect from potential perturbations of the TEM camera length. These normalized FWHM values, which correspond to the spread of the first nearest-neighbor atomic distances [37,38], are shown in Figure 4(c) for sample 2. The FWHM values decreased linearly with increasing stress in both cycles, but saturated at high stresses in the second one (Figure 4(c)). Interestingly, the FWHM versus stress curves for the two cycles do not overlap as the FWHM increased after unloading. It is pertinent to note that the second cycle of straining for sample 2 was carried out after allowing the sample to relax for a period of 100 hrs. The same trend of decreasing FWHM with increasing stress was obtained with the other two samples as well.

### 3.4. Stress–strain response from strain rate experiments

**Figure 5.**
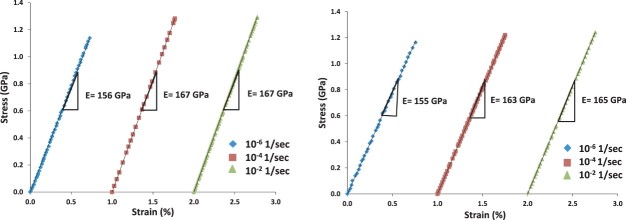
Strain rate dependent Young’s modulus of TiAl films: (a, b) Stress–strain response of two TiAl thin film samples subjected to ex situ straining at three different strain rates. The *E* obtained at the highest strain rate is about 7% greater than the value obtained from the lowest rate. The strain values for the experiments carried out at 10^−4^/s and 10^−2^/s have been offset by 1 and 2% strains, respectively. The errors in stress and strain measurements were ±5 MPa and ±0.01%, respectively.

As mentioned earlier, there was a significant difference between the atomic-level longitudinal elastic strain and macroscopic strain in the *in situ* TEM experiments. To explore the cause of this discrepancy, we conducted *ex situ* tensile load–unload experiments at different strain rates (10^−6^/s–10^−2^/s) on two samples. The strain rates were chosen such that the lowest strain rate was roughly similar to the averaged strain rate during the *in situ* TEM experiments. The experiments showed that the stress–strain response was linear during both loading and unloading at all rates. There was also no residual strain after unloading in any of the experiments, confirming the lack of plastic deformation. However, the measurements revealed a rate dependence of *E* (Figure 5), which increased from 156 ± 1 GPa at the lowest strain rate to 166 ± 1 GPa (6–7% increase) at the highest strain rate (10^−2^/s). Thus, for a given stress, the measured *ε_macro_*_­_ at the highest rates was about 6–7% smaller than the strain at the lowest rate.

## 4. Discussion

The *in situ* TEM experiments of the TiAl films revealed failure stresses between 2.7 and 2.9 GPa and macroscopic failure strains between 1.7 and 1.9%. By tracking the geometric changes in the SAD patterns, we were able to measure the atomic-level elastic strain tensor, which showed that the longitudinal elastic strain (*e_11_*) was consistently smaller by about 10% compared to *ε_macro_*. No discernable plasticity was seen in any of the samples. Nevertheless, an analysis of the atomic-scale response and comparison of the macroscopic strain and atomic-level elastic strain reveals several interesting details about the structural changes induced by the deformation.

First, we consider the discrepancy between *ε_macro_*_­_ and *e_11_* in the amorphous TiAl film. The change in the geometry of the SAD patterns, from which we obtain *e_11_*, is sensitive only to elastic strain [17–18]. In the *in situ* TEM experiments, *ε_macro_*_­_ was about 10% larger than *e_11_*. The strain rate experiments (Figure 5) show that *ε_macro_*_­_ at the lowest rate is 6–7% higher than *ε_macro_*_­_ at the highest rate for a given stress. In effect, at least 6–7% of *ε_macro_*_­_ is due to anelastic deformation at rates (∼10^−6^/s) comparable to those applied in the *in situ* TEM experiments. Thus, a major part of the observed difference between *ε_macro_*_­_ and *e_11_* in the *in situ* TEM experiments can be accounted for by anelasticity.

Furthermore, it has been shown that in metallic glasses, atomic-level elastic strain in the nearest-neighbor shells can be smaller than that in distant atomic shells [18]. Since the geometric changes in the first diffraction ring correspond to the nearest-neighbor shell, it is possible that the calculated *e_11_* underestimates the elastic strain in the film. This could, in principle, explain why anelasticity does not fully account for the difference between *ε_macro_*_­_ and *e_11_*.

In this context, it is worth noting that even in previous *in situ* XRD deformation studies on metallic glasses, *E* calculated using atomic-level elastic strain was about 5–6% greater than the value obtained using macroscopic strain [17,18]. Our results suggest that those differences might also have been the result of anelasticity. Anelastic deformation in metallic glasses is often attributed to the presence of atomic free-volume zones [39], the extent of which depends on the processing conditions and the thermal/mechanical history. Metallic glass thin films that are synthesized by sputtering are known to have a larger fraction of free-volume zones [40], and this could be the reason for the higher anelastic strain observed in our samples.

From a microscopic viewpoint, anelasticity can be seen as a manifestation of deformation-induced structural rearrangements [41,42] and MD simulations have found correlations between the short-range order of atomic clusters and the extent of their anelastic deformation [43]. Our FWHM measurements support such an atomic-scale interpretation of anelasticity. As shown in Figure 4(c), the normalized FWHM values of the amorphous ring decrease with increasing stress, which suggests a change in short-range order (narrower spread of first nearest-neighbor distances). More importantly, the results indicate that these structural rearrangements start to occur from the initial stages of deformation, which is consistent with the presence of anelastic deformation even at low stresses/strains. The saturation of the FWHM values at very high stresses (>2 GPa), however, indicates that the structural rearrangements become progressively harder as the sample approaches catastrophic failure. It is also worth noting that upon unloading after the first cycle, the normalized FWHM increased to a higher value over a period of 100 hrs (Figure 4(c)). One possible reason for this change is the relaxation of internal stresses in the film over time after unloading. More importantly, this change in FWHM suggests that even in the absence of plasticity, the deformation resulted in a permanent change in the amorphous structure of the film.

In addition to anelasticity, the elastic properties (*E* and *µ*) obtained from the *in situ* TEM experiments also provide some insights into the atomic-scale response. Notably, the macroscopic *E* (155 ± 3 GPa) of the metallic glass TiAl film is lower than the *E* of crystalline TiAl (175-188 GPa) due to the presence of anelastic deformation. However, the *E* (173 ± 3 GPa) obtained from the atomic-level longitudinal elastic strain (*e_11_*) is nearly identical to that of crystalline TiAl. This suggests that the atomic-level stiffness of metallic bonds in amorphous TiAl is very similar to that of crystalline TiAl. Similarly, the relatively low Poisson's ratio (*µ* = 0.215 ± 0.02) of the TiAl metallic glass film is also consistent with small failure strains (∼2%). Previous studies have shown that *µ* < 0.3 in metallic glass systems leads to a brittle failure [44] and the lack of plasticity in our samples supports this conclusion.

## 5. Conclusions

Most prevalent techniques for probing the deformation behavior of metallic glasses employ *in situ* high-energy X-ray or neutron diffraction. Here, we have used TEM electron diffraction to calculate the local, atomic-level elastic strain and detect structural rearrangements in nanoscale metallic glass films. By simultaneously measuring the macroscopic stress and strain using a MEMS testing device, we calculated the elastic properties (*E* and *µ*) of amorphous TiAl films and verified those measurements using independent *in situ* SEM experiments. More importantly, we were able to decouple atomic-level elastic strain from anelastic strain, and directly correlate the anelasticity with atomic-scale rearrangements using FWHM measurements of the amorphous ring. The results demonstrate the capability of *in situ* electron diffraction to detect mechanically induced structural rearrangements and quantitatively measure anelastic deformation in nanoscale metallic glass films without the need for experiments at multiple strain rates or frequencies.

## Supplementary Material

Supplementary_file.pdf
