# Supplementary material for: Revealing anelasticity and structural rearrangements in nanoscale metallic glass films using in situ TEM diffraction
Source: Mater Res Lett. 2016 Sep 22;5(3):135–43. doi: 10.1080/21663831.2016.1228709 (PMC5356196; doi:10.1080/21663831.2016.1228709)
Supplement: Supplementary_file.pdf [file tmrl_a_1228709_sm9241.pdf]

Revealing anelasticity and structural rearrangements in nanoscale metallic glass films using in situ TEM diffraction

Rohit Sarkar[1], Christian Ebner[2], Ehsan Izadi[3], Christian Rentenberger[2] and Jagannathan Rajagopalan[1, 3*]

1. Department of Materials Science and Engineering, School for Engineering of Matter, Transport and Energy, Arizona State University, Tempe, AZ 85287, USA.

2. Physics of Nanostructured Materials, Faculty of Physics, University of Vienna, Boltzmanngasse 5, 1090 Vienna, Austria.

3. Department of Mechanical and Aerospace Engineering, School for Engineering of Matter, Transport and Energy, Arizona State University, Tempe, AZ 85287, USA.

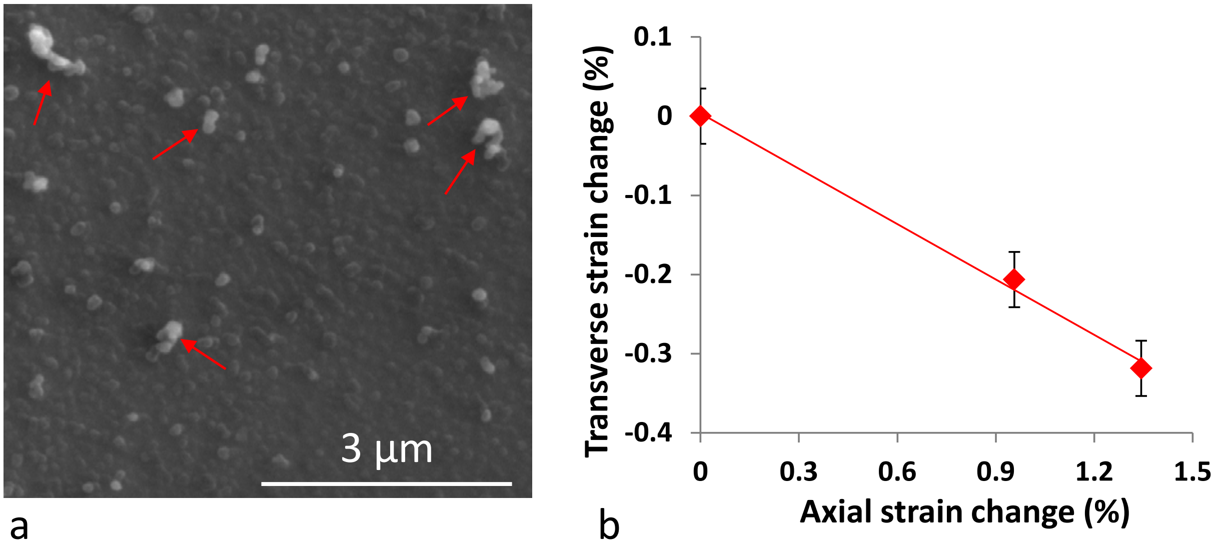

***Supplementary Figure 1: In situ SEM straining experiments**: (a) SEM image of a TiAl sample with some of the residual photoresist markers from sample fabrication indicated by red arrows. (b) Change in transverse strain as a function of axial strain for the sample. The slope of the red line is ~ 0.235, which corresponds to the Poisson's ratio of the sample*

[*]Corresponding author. Email: jrajago1@asu.edu, Phone no: (480) 965-4363
